# The Effect of a Sepsis Interprofessional Education Using Virtual Patient Telesimulation on Sepsis Team Care in Clinical Practice: Mixed Methods Study

**DOI:** 10.2196/35058

**Published:** 2022-04-18

**Authors:** Wei Ling Chua, Sim Leng Ooi, Gene Wai Han Chan, Tang Ching Lau, Sok Ying Liaw

**Affiliations:** 1 Alice Lee Centre for Nursing Studies National University of Singapore Singapore Singapore; 2 Emergency Medicine Department National University Hospital National University Health System, Singapore Singapore Singapore; 3 Yong Loo Lin School of Medicine National University of Singapore Singapore Singapore; 4 Division of Rheumatology Department of Medicine National University Health System, Singapore Singapore Singapore

**Keywords:** sepsis, interprofessional education, team training, nurse-physician communication, simulation, telesimulation

## Abstract

**Background:**

Improving interprofessional communication and collaboration is necessary to facilitate the early identification and treatment of patients with sepsis. Preparing undergraduate medical and nursing students for the knowledge and skills required to assess, escalate, and manage patients with sepsis is crucial for their entry into clinical practice. However, the COVID-19 pandemic and social distancing measures have created the need for interactive distance learning to support collaborative learning.

**Objective:**

This study aimed to evaluate the effect of sepsis interprofessional education on medical and nursing students’ sepsis knowledge, team communication skills, and skill use in clinical practice.

**Methods:**

A mixed methods design using a 1-group pretest-posttest design and focus group discussions was used. This study involved 415 undergraduate medical and nursing students from a university in Singapore. After a baseline evaluation of the participants’ sepsis knowledge and team communication skills, they underwent didactic e-learning followed by virtual telesimulation on early recognition and management of sepsis and team communication strategies. The participants’ sepsis knowledge and team communication skills were evaluated immediately and 2 months after the telesimulation. In total, 4 focus group discussions were conducted using a purposive sample of 18 medical and nursing students to explore their transfer of learning to clinical practice.

**Results:**

Compared with the baseline scores, both the medical and nursing students demonstrated a significant improvement in sepsis knowledge (*P*<.001) and team communication skills (*P*<.001) in immediate posttest scores. At the 2-month follow-up, the nursing students continued to have statistically significantly higher sepsis knowledge (*P*<.001) and communication scores (*P*<.001) than the pretest scores, whereas the medical students had no significant changes in test scores between the 2-month follow-up and pretest time points (*P*=.99). A total of three themes emerged from the qualitative findings: greater understanding of each other’s roles, application of mental models in clinical practice, and theory-practice gaps. The sepsis interprofessional education—particularly the use of virtual telesimulation—fostered participants’ understanding and appreciation of each other’s interprofessional roles when caring for patients with sepsis. Despite noting some incongruities with the real-world clinical practice and not encountering many sepsis scenarios in clinical settings, participants shared the application of mental models using interprofessional communication strategies and the patient assessment framework in their daily clinical practice.

**Conclusions:**

Although the study did not show long-term knowledge retention, the use of virtual telesimulation played a critical role in facilitating the application of mental models for learning transfer and therefore could serve as a promising education modality for sepsis training. For a greater clinical effect, future studies could complement virtual telesimulation with a mannequin-based simulation and provide more evidence on the long-term retention of sepsis knowledge and clinical skills performance.

## Introduction

### Background

Sepsis is defined as a “life-threatening organ dysfunction caused by a dysregulated host response to infection” [1]. Delays in sepsis recognition and slow initiation of treatment have been associated with poor patient outcomes [2,3]. A recent global burden of disease study on sepsis estimated approximately 48.9 million cases of sepsis with 11.0 million sepsis-related deaths worldwide in 2017 [3]. One key effort stipulated by the World Health Organization, which aims to reduce the global burden of sepsis, is to educate health care professionals on the early identification and management of sepsis [4].

As a time-critical illness, early identification, prompt escalation of care, and immediate treatment initiation for sepsis are critical to minimize patient deterioration and improve patient outcomes [3]. Although sepsis is usually managed in intensive care units, there has been an observed increase in the prevalence of sepsis in general wards [5,6]. Thus, nursing and medical graduates must have adequate knowledge and skills to recognize and initiate appropriate management of sepsis because they are typically the first contact point with patients in general wards. Hence, for undergraduate medical and nursing students’ entry into clinical practice, it is important to equip them with the adequate knowledge and skillset required to assess, recognize, escalate care of, and manage patients with sepsis. However, existing literature has revealed that medical and nursing students are underprepared in relation to sepsis recognition and management, suggesting inadequate and ineffective coverage of sepsis education in undergraduate medical and nursing curricula [7-11]. Thus, there is a need to design sepsis education programs that adequately prepare medical and nursing students for entry-level practice.

Successful management of sepsis hinges not only on prompt recognition and immediate response with appropriate escalation of care to critical care if required but also on effective physician-nurse collaboration [12]. Nurses at the bedside play a key role in assessing and recognizing early signs and symptoms of sepsis and then escalating promptly for medical review, whereas junior physicians play a crucial role in considering the possibility of sepsis, initiating prompt initial management, and escalating in a timely manner for intensive care unit care. During this process, interprofessional teamwork and communication are integral to coordinating patient care and delivering timely medical treatment. Therefore, an educational approach that integrates sepsis education and interprofessional team training would further enhance health care professionals’ knowledge and practice of sepsis care. Moreover, the call for interprofessional team training at the prelicensure level makes a strong case for incorporating team training elements, such as interprofessional communication and teamwork, into undergraduate interprofessional education (IPE) programs [13].

Simulation is a popular teaching method to deliver health care team–based training because it provides learners with experiential opportunities to work together as a multidisciplinary team to manage patient care and develop a shared understanding of each other’s roles within a team in regard to patient care [14]. However, logistical challenges, such as conflicting schedules among learners from different professional groups, the availability of facilities and facilitators, and the high cost involved, impede the implementation of in-person simulation–based team training [15]. As such, health care educators have turned to computer learning technology to enhance cost-effectiveness and overcome the barriers to traditional in-person simulation training [16]. Furthermore, restrictions of the COVID-19 pandemic and safe distancing measures have disrupted conventional in-person simulation training [17,18]. This has resulted in an unprecedented push for the adoption of interactive distance learning techniques, such as telesimulation and virtual simulation, to remotely provide simulation-based education [18].

Retaining the experiential strengths of simulation-based learning, telesimulation utilizes both internet-based communication technology and simulation resources to provide simulation-based education when learners and facilitators are at off-site locations [19]. Currently, most telesimulations are hosted on 2D video conferencing software with webcams and screen sharing functions [18]. Facilitators simulate a patient encounter by projecting prerecorded videos of physical examination findings and patient monitors that the facilitators can control based on the clinical scenario flow and participants’ actions [18]. Conversely, virtual reality simulation uses immersive technology to create a 3D computer-based simulation that mimics real-life clinical situations in a virtual environment and can also provide real-time responses according to participants’ actions and decision-making [20,21]. Research has shown that virtual simulation–based team training can improve knowledge retention, clinical reasoning, teamwork attitudes, and communication skills performance, as well as learners’ satisfaction with learning [22-26]. This suggests that virtual simulation is a promising learning strategy for interprofessional team–based learning.

### Objectives

Given that sepsis care is intrinsically interprofessional, requiring input from various health care professionals [12], the combination of IPE and sepsis care provides dual benefits and joint synergy in teaching two important aspects of contemporary health care practice: interprofessional collaborative practice and sepsis care principles. Using a blended learning approach that incorporates virtual telesimulation, we designed and implemented a sepsis IPE program that fulfills the needs of both practical sepsis education and IPE for undergraduate medical and nursing students. An earlier study described the integration of an IPE program into undergraduate medical and nursing curricula using the implementation science method [27]. This study aimed to (1) examine the effects of sepsis IPE using virtual telesimulation on sepsis knowledge and team communication skills of medical and nursing students and (2) describe students’ perceived impact of sepsis IPE on their clinical practice.

## Methods

### Study Design

A mixed methods study design was used, combining a 1-group pretest-posttest design and focus group discussions (FGDs) to evaluate students’ perceived effects of sepsis IPE using virtual telesimulation.

### Study Setting and Participants

To substitute for in-person interprofessional learning during the COVID-19 pandemic, a virtual sepsis IPE program was integrated into undergraduate third-year nursing and fourth-year medical curricula at the National University of Singapore. A total of 96% (288/300) of medical students and 98% (293/299) of nursing students attended this compulsory program. As part of the students’ learning process, all students were required to complete presimulation and postsimulation quizzes.

It was made known to the students that evaluation research would be conducted to evaluate the sepsis IPE program, and participation in the research was completely voluntary. Before starting the sepsis IPE program, a participant information sheet explaining the purpose of the research and outlining the entire research process was sent via email to all students. They were asked to provide consent for the use of their presimulation and postsimulation quiz results for the evaluation research, as well as consent to be contacted to complete a 2-month follow-up test and participate in a one-time FGD after completion of the program.

### Sepsis IPE

#### Presimulation Activities

As part of the presimulation learning activities, the participants attended didactic e-learning on team communication skills strategies and sepsis education on their own time. The learning involved team communication skills strategies adapted from the Team Strategies and Tools to Enhance Performance and Patient Safety curriculum [28], which included the Identity, Situation, Background, Assessment and Recommendation (ISBAR) communication tool; the Concerned, Uncomfortable, and Safety (CUS) tool; and feedback to acknowledge, call out, and check back. The sepsis education adopted a case study teaching method that focused on adult sepsis pathophysiology and clinical manifestations, risk factors of sepsis, assessment of sepsis using the Airway, Breathing, Circulation, Disability, Exposure (ABCDE) approach, diagnostic and laboratory investigations for sepsis, and general management of sepsis and septic shock based on the surviving sepsis campaign guidelines [29]. References to further essential web-based readings were also provided.

#### Virtual Telesimulation

Table 1 summarizes the virtual telesimulation implementation process. Participants were scheduled to participate remotely in a 3-hour virtual telesimulation that we developed using the Unity 5 game engine (Unity Technologies) [30]. The design and learning activities in the sepsis virtual telesimulation were underpinned by the experiential learning theory by Kolb [31] and the theory of social constructivism by Vygotsky and Cole [32]. The experiential learning theory by Kolb [31], which constitutes the learning process of gaining knowledge from experimentation followed by reflection, informed the learning mechanisms of role-playing and debriefing in the virtual telesimulation. The approach of medical-nursing student collaborative learning through role-playing and debriefing supports the theory of social constructivism, which emphasizes learning through interpersonal interaction and discussion [32].

For each session, 2 medical students and 2 nursing students were assigned to a group, and each group included a nursing facilitator or debriefer and a simulated patient. The virtual telesimulation was implemented over a period of 4 months, from August to December 2020, with approximately 150 sessions conducted. In total, 28 clinicians who were nursing alumni of the university and had at least three years of clinical practice experience were recruited as facilitators for the simulation. Every facilitator had to undergo a one-time training session that covered the program’s learning objectives, lesson plans, simulation scenarios, and facilitation and debriefing pointers and instructions on how to navigate in the virtual environment.

**Figure 1:**
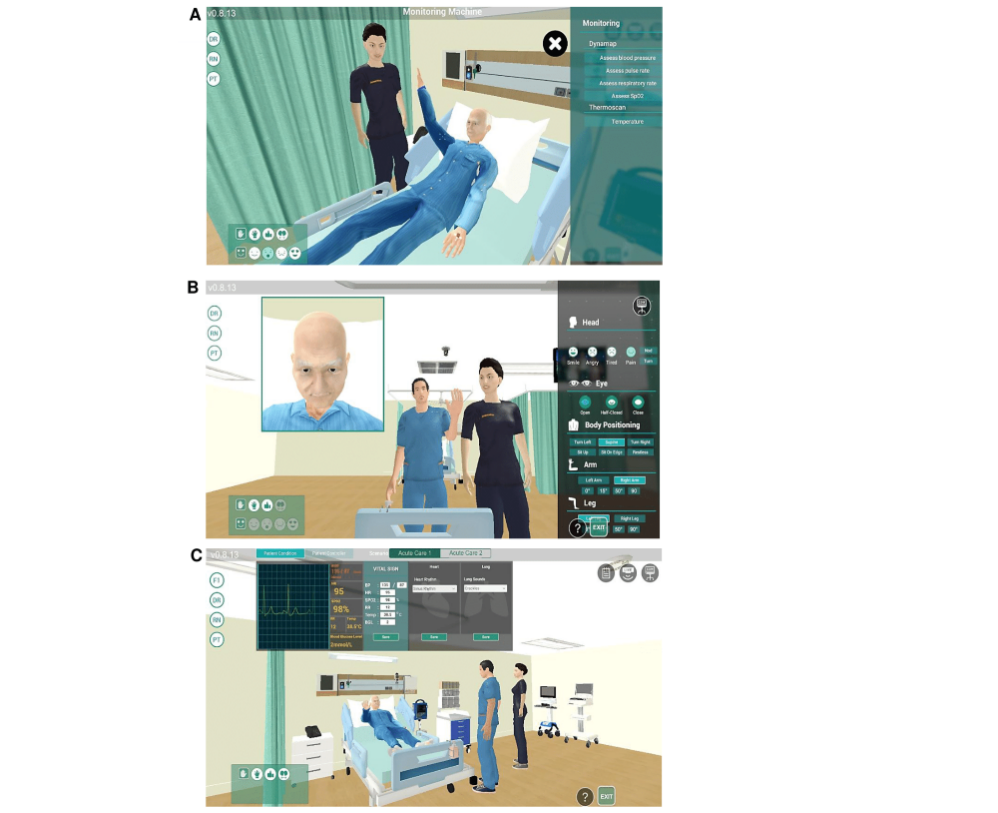
Viewpoints of different avatar roles: (A) nursing or medical student’s view, (B) simulated patient’s view, and (C) facilitator’s view during role-playing.

The virtual telesimulation required only standard computer equipment, and instructions were given to all students to install the virtual simulation software before the telesimulation. At the start of each session, the students were oriented by the facilitator on the Zoom videoconferencing software. During the orientation, the students learned how to navigate in the 3D virtual environment using their human-controlled avatar roles and perform assessments and management on the patient avatar. Both the players (ie, medical and nursing students) and the facilitator can use the computer’s keyboard or mouse to freely navigate inside the virtual hospital and verbally communicate with one another and the simulated patient in real time using headsets or earphones with a microphone. Figure 1 illustrates the views presented to the different avatar roles.

**Table 1:** Technical and educational components of Sepsis IPE^a^ virtual telesimulation.

Task and personnel			Technology		Process
**Simulation orientation**					
	Facilitator and technical support staff	Zoom (Zoom Video Communications, Inc)		Welcome students Introduce team: facilitator and students Reinforce confidentiality and ground rules Learn how to navigate in the virtual environment using avatar roles and testing of audio system for communication Assign students into medical-nursing pair	
**Simulation**					
	Facilitator	Unity 5 games engine		Introduce scenario Allow student players to read case scenario	
	Simulated patient	Zoom		Responding or answering to questions	
	Students (in team 1 medical-nursing pair)	Unity 5 games engine		Perform patient assessment of the patient avatar Initiate interventions and treatments by clicking on the treatment trolley or equipment Communicate with each other and patient avatar using headsets and clickable gestures	
**Debriefing with students**					
	Facilitators	Zoom		Announce end of simulation scenario and instruct students to return to Zoom for debriefing Instruct everyone to turn on video function Engage students in reflection during scenario and from the sepsis IPE	

^a^IPE: interprofessional education.

The 4 students in each group were randomly paired to form 2 medical-nursing student pairs. Each medical-nursing student pair took turns as role-players and observers in the 2 simulation scenarios. The first scenario simulated a postoperative patient with early manifestations of sepsis, which required the team to perform a clinical assessment of patients with suspected sepsis and early goal-directed therapy for sepsis, including oxygen therapy, septic workup, and intravenous antibiotic therapy. The second scenario simulated the same patient who had deteriorated and required airway management and fluid resuscitation.

Before the start of the simulation scenario, each medical-nursing student pair was given 15 minutes to read the case history. Thereafter, each scenario began with the nursing student assessing the patient using the ABCDE approach and initiating immediate nursing management before escalating to their medical teammate. The medical student was expected to perform a patient assessment and collaborate with the nursing student on the treatment plans. They were also expected to use the communication strategies (eg, ISBAR, CUS, and the feedback to acknowledge, check back, and call out strategy) to communicate with each other.

Each simulation scenario lasted approximately 15 to 20 minutes and was followed by a 30-minute semistructured reflective debriefing conducted by a facilitator on the Zoom videoconferencing software. The students were asked to reflect on their performance and discuss the assessment and management of sepsis and septic shock, as well as the process of nurse-physician teamwork, communication, and collaboration. Upon the conclusion of the 2 scenarios, the participants returned to the Zoom videoconferencing software for a team debriefing. The facilitators first asked the participants about their overall thoughts and impressions of the virtual telesimulation. They then reviewed the main clinical knowledge and teamwork learning points and ended with each learner citing their major take-home points from the sepsis IPE.

### Data Collection

Both quantitative and qualitative data were obtained in this study. Whereas the quantitative data focused on knowledge evaluation and retention of the sepsis IPE, the qualitative data aimed to capture the participants’ perspectives on the transferability of learning to clinical practice.

Quantitative outcomes were collected at three time points using the same set of quizzes: before (pretest), immediately after (posttest), and 2 months after (follow-up) the virtual simulation. The quiz consisting of 4 short case studies on sepsis with 25 multiple-choice questions was designed by the study team to assess sepsis knowledge (18 questions) and team communication skills (7 questions; Multimedia Appendix 1). The quiz topics were covered in the e-learning modules and virtual simulation. The quiz questions were developed with reference to the Surviving Sepsis Campaign Guidelines for Management of Sepsis and Septic Shock 2016 [33] and Team Strategies and Tools to Enhance Performance and Patient Safety curriculum [28]. The quiz was content validated by a multidisciplinary team consisting of 1 medical doctor, 2 nursing academics, and 1 advanced practice nurse, who were involved in undergraduate medical and nursing teaching programs.

Qualitative data were collected through FGDs. A total of 4 focus groups were conducted approximately 5 months after the completion of the sepsis IPE. They were conducted by a moderator on Zoom using a semistructured topic guide (Textbox 1), and field notes were taken by a research assistant. The moderator was a research fellow who did not have any previous interaction with the participants. This helped minimize any influence of the moderator on the participants during the FGDs. Each focus group consisted of 4 to 6 participants from the same profession and lasted 50 to 75 minutes. All FGDs were audio recorded.

**Focus group discussion topic guide.**

**Recall of sepsis interprofessional education (IPE) learning**

- What have you learned from the sepsis IPE program?
- What are your thoughts about this interprofessional sepsis care training? In what ways were it effective?
- Which aspect of your knowledge and action skills on sepsis care do you think has improved?

**Application of learning in clinical practice**

- Since the end of the sepsis IPE program, have you had any opportunity to apply any of the knowledge or skills learned in clinical practice? Please share.
- Tell me about your experience and level of comfort with using the communication strategies?
- Has the program changed your way of communication with other health care professionals in clinical setting?
- What are some barriers to the application or transfer of learning to clinical practice?

**Areas of improvement for future sepsis IPE program**

- How can we improve future sepsis IPE program?
- Do you think it is necessary to have interdisciplinary facilitators for the virtual simulation?

### Data Analysis

All quantitative data were analyzed using IBM SPSS Statistics for Windows (version 26.0; IBM Corp) [34]. Descriptive statistics were used to summarize the participants’ demographics and sepsis knowledge and team communication scores at each time point. A 2-tailed, paired sample *t* test was used to examine changes between the baseline and immediate posttest scores, whereas repeated-measures ANOVA with Bonferroni correction was used to determine the effect of the intervention over the 3 time points, as well as on the respective profession group. For all analyses, the level of statistical significance was set at *P*<.05.

The audio-recorded FGDs were transcribed verbatim and subjected to the thematic analysis of Braun and Clarke [35]. Field notes were inserted into the transcripts. In addition, 2 investigators independently coded the transcripts and generated emerging themes before comparing and discussing the final set of themes and subthemes. Thematic saturation was deemed to have been achieved through the 4 FGDs.

### Ethical Considerations

This study was approved by the National University of Singapore Institutional Review Board (Reference Code S-17-107). Informed consent for voluntary participation was obtained from all participants. Furthermore, participants were assured of confidentiality, anonymity, and their right to withdraw from the study at any time without any repercussions. However, they were informed that the data that had been collected until the time of their withdrawal would be retained and analyzed to enable a complete and comprehensive evaluation of the study.

## Results

### Effect of Sepsis IPE on Sepsis Knowledge and Team Communication

Of the 581 students who attended the program, 415 students consented to participate in the sepsis IPE research study. A total of 214 (73%) out of 293 nursing students and 201 (69.8%) out of 288 medical students who attended the program consented to participate in the research study. All 415 participants completed the presimulation and postsimulation tests. The medical students had statistically significant higher pretest scores for both sepsis and communication than nursing students (Table 2). After attending the sepsis IPE, the medical students continued to have significantly higher posttest scores than the nursing students, except for communication scores (*P*=.32). Nonetheless, as shown in Table 3, both the medical and nursing students showed significant improvements in their posttest scores for sepsis knowledge and communication after attending the sepsis IPE.

**Table 2:** Summary of mean scores before and after virtual simulation.

Test		Medicine (n=201), mean (SD)	Nursing (n=214), mean (SD)	*P* value
**Pretest**				
	Total score (0-25)	16.69 (2.77)	12.67 (2.99)	<.001
	Sepsis knowledge (0-18)	12.33 (2.14)	8.82 (2.22)	<.001
	Communication (0-7)	4.34 (1.59)	3.85 (1.59)	.002
**Posttest**				
	Total score (0-25)	19.09 (2.41)	16.30 (2.90)	<.001
	Sepsis knowledge (0-18)	13.56 (1.68)	10.90 (2.20)	<.001
	Communication (0-7)	5.53 (1.46)	5.40 (1.40)	.32

**Table 3:** Comparison of the pretest and posttest mean scores within the medical and nursing students.

Test			Total score (0-25)		Sepsis knowledge (0-18)		Communication (0-7)
**All participants (N=415)**							
	Pretest, mean (SD)	14.62 (3.51)		10.52 (2.80)		4.09 (1.60)	
	Posttest, mean (SD)	17.66 (3.01)		12.19 (2.37)		5.47 (1.43)	
	*P* value	<.001		<.001		<.001	
**Nursing (n=214)**							
	Pretest, mean (SD)	12.67 (2.99)		8.82 (2.22)		3.85 (1.59)	
	Posttest, mean (SD)	16.30 (2.90)		10.90 (2.20)		5.40 (1.40)	
	*P* value	<.001		<.001		<.001	
**Medicine (n=201)**							
	Pretest, mean (SD)	16.69 (2.77)		12.33 (2.14)		4.34 (1.59)	
	Posttest, mean (SD)	19.09 (2.41)		13.56 (1.68)		5.53 (1.46)	
	*P* value	<.001		<.001		<.001	

### 2-Month Follow-up Results

A total of 35% (75/214) of nursing students and 24.9% (50/201) of medical students completed the 2-month follow-up test. The Bonferroni test indicated that there were significant differences in the students’ knowledge levels across time. The post hoc comparisons of the presimulation and 2-month follow-up test scores and postsimulation and 2-month follow-up test scores within each group are provided in Table 4.

At 2-month follow-up, the nursing students continued to have statistically significantly higher sepsis knowledge (mean 10.16, SD 2.42) and communication scores (mean 5.0, SD 1.55) than the pretest scores (sepsis knowledge: mean 9.21, SD 1.93; communication: mean 3.91, SD 1.56). For the medical students, there was a significant increase in sepsis knowledge test scores and communication scores from the presimulation test to the postsimulation test (sepsis knowledge: mean 12.26 SD, 1.94 vs mean 13.56, SD 1.95, *P*<.001; communication: mean 4.66 SD, 1.53 vs mean 5.60, SD 1.68, *P*<.001), but no significant changes in test scores between the 2-month follow-up and presimulation tests. Nevertheless, the medical students continued to have significantly higher sepsis knowledge scores at 2 months’ follow-up than the nursing students (mean 12.38, SD 1.97 v mean 10.16, SD 2.42, *P*<.001). Although there was no significant difference in the communication scores between the medical and nursing students (*P*=.37), the change between the pretest scores and 2-month follow-up test scores was higher among the nursing students (nursing: mean 1.09, SD 1.89, *P*<.001 vs medicine: mean 0.060, SD 2.316, *P*=.86).

**Table 4:** Comparison of pretest-posttest scores and 2-month follow-up test scores.

Test			Nursing (n=75)							Medicine (n=50)				
			Value, mean (SD)		Follow-up, mean (SD)		*P* value		Value, mean (SD)			Follow-up, mean (SD)		*P* value
**Total score**														
	Pretest	13.12 (2.66)		15.07 (3.05)		<.001^a^		17.18 (3.31)			17.10 (2.38)		.99^a^	
	Posttest	16.89 (3.13)		15.07 (3.05)		<.001^b^		19.16 (2.87)			17.10 (2.38)		.001^b^	
	*P* value	<.001^c^		N/A^d^		N/A		<.001^c^			N/A		N/A	
**Sepsis knowledge**														
	Pretest	9.21 (1.93)		10.16 (2.42)		.008^a^		12.26 (1.94)			12.38 (1.97)		.99^a^	
	Posttest	11.39 (2.32)		10.16 (2.42)		.001^b^		13.56 (1.95)			12.38 (1.97)		.001^b^	
	*P* value	<.001^c^		N/A		N/A		.002^c^			N/A		N/A	
**Communication**														
	Pretest	3.91 (1.56)		5.00 (1.55)		<.001^a^		4.66 (1.53)			4.72 (1.90)		.99^a^	
	Posttest	5.51 (1.52)		5.00 (1.55)		.04^b^		5.60 (1.68)			4.72 (1.90)		.01^b^	
	*P* value	<.001^c^		N/A		N/A		.02^c^			N/A		N/A	

^a^Comparison of 2-month follow-up test scores with pretest scores.

^b^Comparison of 2-month follow-up test scores with posttest scores.

^c^Comparison of pretest scores with posttest scores.

^d^N/A: not applicable.

### Effect on Clinical Practice

#### Overview

A total of 10 medical and 8 nursing students participated in 4 FGDs. Among the participants, there were 4 male medical students and 1 male nursing student. The thematic analysis yielded three main themes: (1) greater understanding of each other’s roles, (2) application of learning in clinical settings, and (3) theory-practice gaps.

#### Theme 1: Greater Understanding of Each Other’s Role

Generally, both the medical and nursing students agreed that the use of sepsis case scenarios was suitable for interprofessional team–based training because it provided them with a greater understanding and appreciation of each other’s interprofessional roles when caring for patients with sepsis:

Sepsis scenario allows medical and nursing students to work together because it is a condition that requires team effort to treat the patient.

Nursing FGD1, P2

I think it [sepsis IPE learning] was a good experience to learn from our nursing colleagues. It helped me see what they [nurses] focus on and their thought process before deciding whether to escalate to the doctors and what information that they chose to pass down that they felt was important...also more of understanding the roles of both the doctors and nurses.

Medical FGD2, P2

Participants also stated that the use of virtual telesimulation and gamification was not only refreshing in fostering the process of interprofessional teamwork and communication in the management of septic patients but also assisted with the application of knowledge, as well as knowledge retention:

I thought it [sepsis virtual telesimulation] was quite fun...I learnt how to manage sepsis and how to communicate with the nurses, and then use it [acquired knowledge] in the game itself. I also got to understand what the nurses could do in their capacity, [and have a] better understanding of their roles and the team.

Medical FGD1, P4

I think having a virtual telesimulation with regards to sepsis would definitely help us to retain the information better. If without the telesimulation, we will just be reading off slides and textbooks, so the retention is definitely not as good as when we have experienced it in a virtual setting or in a real-life setting.

Nursing FGD2, P5

Generally, participants were satisfied with having just a nursing facilitator to facilitate the sepsis IPE. They felt that the nursing facilitators could provide insights into the interprofessional teamwork approach to the medical and nursing management of sepsis that catered to both professions:

My nursing facilitator is very experienced. She knows a lot as to bringing in her nursing experiences as well as some clinical value. I don’t think that a medical facilitator will be essential. Because if there are many people, everyone wants to say something. Then, it becomes a bit more artificial...if we want to learn more about the sepsis and protocol, we can learn from the doctors in the wards.

Medical FGD2, P1

#### Theme 2: Application of Mental Models in Practice

During the FGDs, all participants shared their main takeaways from the program. The nursing participants gained knowledge predominantly on using the ABCDE approach to assess and manage patients with sepsis and effective nurse-physician communication strategies. Following the sepsis IPE program, the nursing students attended their transition-to-practice clinical practicum, which required them to take on the responsibilities of a full-fledged registered nurse under supervision. Although most participants did not encounter sepsis during their clinical practicum, they shared the application of the mental models, including the ABCDE assessment framework and communication strategies, in their daily clinical practice:

For me, learning about the ABCDE assessment part and what to do when patient deterioration happens, was main takeaway.

Nursing FGD1, P1

I don’t think it’s about [knowledge] retention, but application. I’m sure that there are some points that we can apply in other scenarios such as ABCDE assessments and communication strategies.

Nursing FGD2, P3

In contrast, the medical students benefited more from interprofessional teamwork and communication skills. They reported having greater awareness of the use of these communication strategies, although they cited a lack of opportunities to apply the interprofessional communication strategies in clinical settings because the nature of their clinical practicum was to *shadow* the medical team and attend case discussions:

The main takeaway would be the communication strategies because the callout and checkback, they are all new to me until the IPE session. Even though I haven’t got to use it directly as a medical student in the ward, it made me more aware of such communication techniques.

Medical FGD1, P4

Experiencing on a virtual platform that there are these communication strategies, you start to observe this in the clinical settings when you see your seniors speak to nurses or other healthcare professionals. Then, you start to realise that they do use it on day-to-day basis.

Medical FGD2, P1

#### Theme 3: Theory-Practice Gaps

Despite observing the value of the ABCDE framework and communication strategies, the nursing participants highlighted a disconnect between what they were taught and what they practiced in the clinical setting. The nursing participants reported having to adapt the ABCDE and ISBAR frameworks to suit clinical workflows and dynamics. Such instances include nonadherence to the ABCDE framework of patient assessment and modification of ISBAR when escalating to the medical team:

They [ward nurses] know what to do first then they will continue assessing patient. The ABCDE is not always [done] in systematic order...For us [nursing students], I think the ABCDE is quite a good framework. At least you have it at the back of your mind and you know what to do next when your patient deteriorates.

Nursing FGD1, P3

I didn’t apply the full ISBAR. I never give the background of this patient because the doctor always seems to be in a rush, and they don’t really want to hear you talk so much about patient’s background because they can find out themselves afterwards. So, I just give them assessment and what is happening to the patient.

Nursing FGD1, P2

Although both the medical and nursing students welcomed the virtual telesimulation, they also acknowledged the value of physical simulation in terms of its realism and ability for psychomotor skills practice. They suggested that virtual telesimulation should complement and be integrated into their existing curriculum for continuity:

I really appreciate that it was a game because it was more engaging. But I would prefer if we can also do it in real life. I think, in terms of assessing the patients, it is a bit superficial to just click on the buttons. If you do it in real life, there is more practical [hands-on] aspect.

Medicine FGD1, P1

Having it [virtual telesimulation] like a more continuous or regular thing rather than just a one-off thing instead.

Nursing FGD2, P6

## Discussion

### Principal Findings

To the best of our knowledge, this is the first study to evaluate the effect of a sepsis IPE program using virtual telesimulation on the sepsis knowledge and team communication skills of undergraduate medical and nursing students. Our mixed methods approach enabled the evaluation of the program at the second and third levels of Kirkpatrick’s model of training evaluation [36]. Level 2, *learning*, was assessed through the quantitative sepsis knowledge and communication quiz, whereas level 3, *behavior changes*, was evaluated through FGDs on the effect of the sepsis IPE on students’ clinical practice [36].

### Comparison With Previous Work

In this study, we built the students’ knowledge by scaffolding the knowledge base, starting with didactic e-learning, followed by a skills practice session through virtual simulated cases with debriefing sessions. Both the medical and nursing students had improved knowledge acquisition as measured by their sepsis knowledge and communication quiz scores immediately after training. This finding is analogous to those of previous studies [21,37], which observed an immediate measurable improvement in theoretical knowledge with learning through virtual simulation. Through role-playing exercises and engaging in reflective debriefing, the virtual telesimulation provided opportunities for the medical and nursing students to work together in a realistic environment and practice their skills in problem solving, decision-making, and team communication, which are essential for the management of patients with sepsis [21]. This application of experiential learning through virtual telesimulation helped deepen students’ learning from self-directed e-learning by building connections between theory and clinical practices [38].

At 2-month follow-up, both groups had lost knowledge over time. Although this is as expected and aligns well with other findings of retention effects of virtual simulation training in health education [39-41], the benefit of virtual simulation—namely, unlimited opportunities to engage in repetitive practice within a safe and realistic clinical environment [41]—was not maximized in this study. Repetition of virtual simulation sessions is key to the long-term retention of learning [42]. However, the virtual telesimulation was a one-off session in this study, which could have limited the true retention effects of virtual simulation training. Similar to an earlier study by Liaw et al [22], to some extent, the use of multiuser human-controlled avatars posed a challenge to bringing the medical and nursing students together at the same time to form interprofessional teams for regular virtual simulation sessions. The development of embodied virtual agents (EVAs; ie, physicians, nurses, simulated patients, or even facilitators) controlled by computer algorithms to allow for a single-player mode could potentially be a solution to achieve better scalability and sustainability of team-based training; however, further evaluation is needed to compare the effectiveness of EVAs (single-player mode) with human-controlled avatars (multiplayer mode) in virtual team-based simulations [22].

When compared with the preintervention test, the nursing students demonstrated significantly higher sepsis knowledge and communication scores on the 2-month follow-up test. This finding suggests that there is little knowledge decay among the nursing students. However, the medical students did not show significant differences in either sepsis or communication scores when compared with the preintervention test scores, suggesting no knowledge retention. There are 3 plausible explanations for the more positive results among the nursing students. First, medical students had higher mean pretest scores than nursing students, and it was predicted that learners with higher pretest scores would have lower learning gains than learners with lower pretest scores [43]. Second, one could theorize that opportunities for repetitive practice are crucial in enhancing knowledge retention [41]. Shortly after the sepsis IPE, the nursing students completed their final clinical practicum, in which they were expected to function as registered nurses. The requirements of their clinical practicum would have provided them with opportunities to deliberately practice the acquired knowledge, thus aiding knowledge retention. This was corroborated in both the quantitative and qualitative data, whereby the nursing participants continued to have significantly higher communication scores at 2-months follow-up from the preintervention test and reported the application of team communication strategies in their daily clinical practice. Congruently, the significant drop in communication scores at 2 months’ follow-up to a mean score on par with the preintervention test among the medical students could be attributed to a lack of opportunities to practice interprofessional communication. The third reason could be related to the lack of medicine facilitators in the interprofessional sepsis team training. Although the medical students did not feel a compelling need to have a medicine facilitator, including facilitators of the respective professions (medicine or nursing) could enhance students’ learning through the sharing of their respective professional perspectives and practices in real clinical practice [44,45].

Overall, although long-term sepsis knowledge retention may not be apparent in our quantitative data, our qualitative data showed that the sepsis IPE had a positive effect on students’ awareness of sepsis and fostered a better understanding and appreciation of each other’s interprofessional roles. This finding is consistent with those of previous studies [22,23], where significant improvements in attitudes toward health care teams and interprofessional collaboration were observed following interprofessional team–based training using virtual simulation. Similar to conventional in-person interprofessional simulation, virtual telesimulation is an experience-based learning strategy that gives learners an opportunity to experience interprofessional collaborative practice in a realistic and risk-free environment [17,20]. Although this study demonstrated that virtual telesimulation is not inferior to in-person simulations in interprofessional team training, further research is needed to evaluate this educational modality in the development of students’ clinical and team communication skills.

Our method of incorporating mnemonic tools (ie, the ABCDE patient assessment framework and ISBAR communication tool) as mental models into the simulation learning was observed to facilitate the transfer of learning from the sepsis IPE to the clinical setting. Despite the lack of opportunities to practice communication skills in their clinical practice, the medical students were more cognizant of the communication strategies for effective communication within interprofessional health care teams. For the nursing students, the teaching of the systematic ABCDE assessment tool and team communication skills was applied to their everyday practice in the clinical setting. Although the nursing students noted some variability in real-world clinical practice, especially in relation to patient assessment, this inconsistency is not surprising. Instead of taking a step-by-step ABCDE approach to patient assessment, more experienced nurses tend to collect a range of focused and relevant cues to obtain a complete picture of the patient’s situation because they are better able to anticipate the patient’s problems [46]. Conversely, for students who lack clinical exposure, the use of mnemonic tools provides mental models to enhance learning and boost the recall of learned information, which in turn aids in the application of learning to practice [47].

Interestingly, although the value of using virtual telesimulation to deliver the sepsis IPE was well received by the students, both the medical and nursing students were in favor of an additional in-person simulation after the virtual simulation to consolidate and reinforce their learning. This is unsurprising; several studies have shown a strong preference for the kinesthetic learning style among medical and nursing students [48-52]. Despite the aforementioned merits of virtual telesimulation, it is lacking in terms of procedural skill enhancement. For a topic such as sepsis, which involves clinical procedural skills, in-person simulations provide kinesthetic learning in a realistic situation that could better develop procedural skills [53]. Thus, a blended simulation approach (ie, virtual telesimulation followed by high-fidelity mannequin-based simulation) could optimize sepsis care learning because of its ability to provide both formative and summative assessments of knowledge and skills acquisition [38,54].

### Limitations

This study had a few limitations that warrant attention. First, the effectiveness of our sepsis IPE using virtual simulation was limited by the absence of a control group and an evaluation of long-term knowledge retention that was confined to a relatively small sample size (125/415, 30.1%) of students’ knowledge 2 months after the program. The high rate of loss to follow-up was likely due to the students’ heavy workload, as the data collection period coincided with their clinical practicum and midterm tests. Notwithstanding, the FGDs provided some insights into the students’ transfer of learning to the clinical setting. Second, the program was evaluated based on a multiple-choice quiz that assessed the lower levels of clinical competence—that is, the cognition domain of fact gathering and application of knowledge—rather than clinical performance and actual practice, which are typically evaluated using a simulation test with an assessor checklist or workplace-based assessment [55,56]. Third, our evaluation may be subject to recall or practice effect bias because we used the same set of quizzes to evaluate the postsimulation test and follow-up test. It might have been better if we had modified the set of quizzes, but to a similar difficulty level as the presimulation quiz. Furthermore, the FGDs were conducted 5 months after the simulation learning, which might have resulted in erroneous recall of responses from the participants. Finally, the true effect of the program may have been constrained by the one-time exposure to the virtual simulation experience when the intent was to provide greater access to enable students to deliberate practice opportunities.

### Future Directions

Given that the virtual telesimulation was a one-off session in this study, further research with a control group is needed to determine whether exposure to repetitive virtual telesimulation training can mitigate knowledge decay over time and contribute to the long-term retention of clinical competency through high-fidelity mannequin-based simulation assessments. Further development and evaluation of EVAs controlled by computer algorithms to allow for the single-player mode as opposed to the multiplayer mode could address the desire for time flexibility, accessibility to repetitive training, and scalability to train a large number of learners. Future studies could also evaluate the effect of virtual telesimulation followed by high-fidelity mannequin-based simulation on the long-term retention of team performance on sepsis management, as well as evaluate the transfer of students’ learning to clinical practice as junior house officers and new graduate nurses. On a broader context, more study is warranted to evaluate virtual telesimulation as an educational modality on clinical skills development.

### Conclusions

A sepsis IPE program using a virtual simulation was developed to enhance sepsis knowledge and team communication skills among medical and nursing students. Although long-term sepsis knowledge retention was not demonstrated in this study, virtual telesimulation played a critical role in facilitating the application of knowledge and skill utilization in the clinical setting. The study also achieved one of its objectives, namely, strengthening interprofessional collaboration, whereby students fostered a better understanding and appreciation of each other’s interprofessional roles in sepsis care. Future studies could complement the virtual telesimulation with a mannequin-based simulation and provide more evidence on the long-term retention of sepsis knowledge and clinical skills performance.

## Appendix

Multimedia Appendix 1 Sepsis IPE Quiz.

## Abbreviations

ABCDE: Airway, Breathing, Circulation, Disability, Exposure
EVA: embodied virtual agent
FGD: focus group discussion
IPE: interprofessional education
ISBAR: Identity, Situation, Background, Assessment and Recommendation
