# Supplementary material for: The Effect of a Sepsis Interprofessional Education Using Virtual Patient Telesimulation on Sepsis Team Care in Clinical Practice: Mixed Methods Study
Source: J Med Internet Res. 2022 Apr 18;24(4):e35058. doi: 10.2196/35058 (PMC9062715; doi:10.2196/35058)
Supplement: Multimedia Appendix 1 [file jmir_v24i4e35058_app1.pdf]

Multimedia Appendix

SEPSIS IPE QUIZ

Instructions:

1. Answer ALL questions in one session
2. Please do not refer to any reading or learning materials when answering the questions

A. Participant demographic characteristics

A1. Age: ________

A2. Gender: ☐Male ☐Female

A3. Course: ☐Medicine ☐Nursing

Questions 1 – 6 are based on the following case study:

Mdm Lee, a 67-year-old woman, was admitted for her second round of high-dosage chemotherapy for breast cancer. She had a mastectomy 2 months ago and a double-lumen peripherally inserted central catheter (PICC) line inserted at that time. After the chemotherapy session, you observed that Mdm Lee was confused and agitated.

Her vital signs were temperature 37.6°C, heart rate 98 beats/min, respiratory rate 26 breaths/minute, blood pressure 102/53mmHg, and SpO_2 96% on room air.

Physical examination revealed a more reddened and inflamed site of the PICC line than previously.

1. What findings indicate Mdm Leo is in the "early" or "compensated" stage of sepsis?

☐ Tachypnea
☐ Generalised edema
☐ Cool and clammy skin
☐ Temperature of **37.6°C**

2. Using the ISBAR tool to escalate your concerns, which of the following information would you include for the "Assessment" component?

☐ Your concern that patient is confused and vital signs reading.
☐ Baseline data, medical history, and current management.
☐ You suspect that Mdm Lee could have developed PICC line infection.
☐ Condition of the mastectomy site.

3. The following are risk factors that predispose Mdm Lee to sepsis **<u>EXCEPT</u>**

☐ old age
☐ family history
☐ weakened immune system
☐ invasive lines

4. In response to sepsis, Mdm Lee develops vasodilation as manifested by:

☐ cool and clammy skin
☐ generalised edema
☐ capillary refill < 2 seconds
☐ tachypnea and hypotension

5. The likely cause of Mdm Lee's confusion and agitation is due to:

☐ compensatory mechanisms
☐ inflammation of PICC site
☐ decreased cerebral perfusion
☐ side effect of chemotherapy

6. A septic workup includes all of the following **<u>EXCEPT</u>**
☐ arterial blood gas
☐ blood and urine cultures
☐ remove the PICC line and send for culture
☐ serum procalcitonin

Questions 7 – 11 are based on the following case study:

> **Mdm Chin, an 83-year-old woman, was admitted from the nursing home for fever and sudden onset of delirium. She had an indwelling urinary catheter and a stage 3 pressure ulcer over her sacrum. She has a medical history of hypertension and type II diabetes mellitus.**
>
> **On admission, her vital signs were temperature 38.5°C, heart rate 94 beats/min, respiratory rate 28 breaths/min, blood pressure 96/53mmHg, and SpO_2 92% on room air.**

7. Under the ISBAR communication tool, which of the following statement describes the **SITUATION** of Mdm Chin?

   ☐ Mdm Chin needs to be evaluated immediately and may need IV fluid.
   ☐ Mdm Chin has just admitted for fever and sudden onset of delirium and has a stage 3 pressure ulcer over her sacrum.
   ☐ Mdm Chin has low blood pressure of 96/53mmHg and tachypnea.
   ☐ Mdm Chin could have developed sepsis.

8. The doctor acknowledged and thank the nurse for highlighting his/her concern about the change in patient status. Which of the following communication strategy did the doctor apply?

   ☐ call-out
   ☐ check-back
   ☐ concerned, uncomfortable, safety (CUS)
   ☐ feedback

9. You would initiate oxygen therapy for Mdm Chin

   ☐ with the aim of achieving SpO_2 ≥94%.
   ☐ only if her SpO_2 is below 90%.
   ☐ with the aim of ensuring that the SpO_2 does not drop further.
   ☐ only if the underlying cause of her desaturation is related to respiratory failure.

10. In the case of Mdm Chin, which of the following will increase oxygen delivery to her tissues by the greatest delivery?

    ☐ red blood cells transfusion
    ☐ oxygen therapy
    ☐ fluid challenge
    ☐ sodium bicarbonate

11. If Mdm Chin develops sepsis, what would you expect her urine output to be?

☐ <30mls/hr
☐ 30 – 60mls/hr
☐ 60 – 100mls/hr
☐ >100mls/hr

Questions 12 – 18 are based on the following case study:

Mr Yan, a 55-year-old man, was been admitted for pneumonia. He presented with shortness of breath and feeling generally unwell. His wife reported that he had been coughing up brown sputum since the day before. He has medical history of COPD and had recently quitted smoking. His chest x-ray showed bilateral patchy consolidation.

His vital signs were temperature 35.8°C, heart rate 125 beats/min, respiratory rate 30 breaths/min, blood pressure 92/57mmHg, and SpO_2 90% on 40% oxygen. Lung auscultation revealed bilateral crackles over the base of lung.

Physical examination revealed cool, mottled extremities, and capillary refill > 3 seconds. He had not passed urine for the past 4 hours.

12. Your immediate management include all **EXCEPT**:

☐ administer high flow oxygen
☐ escalate for medical review
☐ place patient on at upright position
☐ establish intravenous access

13. Which of the following feature is indicative of an emerging sepsis?

☐ hypotension
☐ oliguria
☐ hypothermia
☐ cool peripheries

14. You use the ISBAR format to communicate a change in patient status to a medical staff. In which order should you make the following statements?

A. The patient needs to be evaluated immediately and may need IV fluid.
B. The patient was admitted last night with pneumonia, with a medical history of COPD.
C. The patient's condition is very unstable and could be having sepsis.
D. I am very concerned about his decreased BP 95/64mmHg and increased heart rate 127 beats/min over the past hour. He has cool and mottled extremities, capillary refill > 3 seconds, and has not passed urine out the past 4 hours as well.

☐ B, C, D, A
☐ C, B, D, A
☐ D, B, C, A
☐ B, D, C, A

15. The doctor gave a verbal order to administer IV fluid stat over the phone. The nurse should administer the IV fluid

☐ only after a written order has been documented in the patient's medical record
☐ immediately without any further delay.
☐ only after the doctor has personally review the patient.
☐ only after he/she has verified the order with the doctor on the phone.

16. What is the first-line IV fluid of choice for sepsis resuscitation?

☐ normal saline 0.9%
☐ dextrose saline
☐ lactate ringers
☐ albumin

17. What is the most likely cause of Mr Yan's hypotension?

☐ decreased blood volume
☐ decreased renal function
☐ compromised cardia contractility
☐ arterial and venous dilatation

18. Which of the following is a biomarker of septic shock?

☐ high white blood cell count
☐ C-reactive protein
☐ lactate level
☐ glucose level

Questions 19 – 25 are based on the following case study:

Mr Tay, a 72-year-old man, was admitted to the ward following a below-knee amputation over the right lower leg due to diabetic foot ulcer. At 2am of postoperative day 5, he developed a sudden fever with a temperature of 38.9°C. heart rate 120 beats/min, respiratory rate 26 breaths/min, blood pressure 88/56mmHg, and SpO2 98% on room air.

On examination, his right lower limb above the amputation stump was cold and had weak pulse. There was also a foul smell from the amputation stump.

19. The priority management for Mr Tan's deteriorating condition is which of the following?

☐ Administer fluid
☐ Administer IV antibiotics
☐ Keep the body temperature below 37.5 C
☐ Monitor electrolytes

20. Which of the following is/are assertive statement(s) that can be used to advocate for doctor's review?

☐ This is a patient safety issue.
☐ I am concerned.
☐ I am uncomfortable.
☐ All of the above

21. Before giving IV antibiotics, which of the following is most important to obtain?
☐ arterial blood gas
☐ blood culture
☐ full blood count
☐ serum lactate

22. Which of the following antibiotics would most likely prescribed for Mr Tay?

☐ rifampicin
☐ piperacillin-tazobactam
☐ linezolid
☐ vancomycin

23. The doctor gave a verbal order to administer IV fluid stat and also communicated the target systolic blood pressure goal for Mr Tay. Which of the following communication strategy did the doctor use?

☐ call-out
☐ check-back
☐ concerned, uncomfortable, safety (CUS)
☐ feedback

24. Mr Tay remains hypotensive despite intravenous fluid resuscitation. He would be classified as having

☐ systemic inflammatory response syndrome
☐ early sepsis
☐ severe sepsis
☐ septic shock

25. Despite giving 2 litres of fluid resuscitation, Mr Tay's blood pressure was still 80/50mmHg, what is the next most appropriate intravenous treatment?

☐ adrenaline
☐ albumin
☐ noradrenaline
☐ lactated ringers
